# Friction of osteoarthritic cartilage with patient-specific synovial fluid: Effect of different loading conditions

**DOI:** 10.1016/j.ocarto.2025.100568

**Published:** 2025-01-16

**Authors:** Luisa de Roy, Jonas Walter Metzger, Martin Faschingbauer, Anita Ignatius, Andreas Martin Seitz

**Affiliations:** a Institute of Orthopedic Research and Biomechanics, Center for Trauma Research, Ulm University Medical Center, Ulm, Germany; b Department of Orthopedic Surgery, RKU, Ulm University Medical Center, Ulm, Germany

**Keywords:** Cartilage, Osteoarthritis, Friction, Synovial fluid

## Abstract

**Objective:**

The objective of this study was to quantify the friction coefficients of degenerated human cartilage lubricated with patient-specific synovial fluid under four different loading regimes in order to identify those regimes that cause the highest friction.

**Method:**

Lateral tibial plateaus and synovial fluid samples were obtained from six patients undergoing total knee replacement surgery. Friction tests were performed on cylindrical samples using an established cartilage against glass tribometer. Four different loading regimes were applied, representing physiologic loads and velocities observed during daily activities. To account for effects of osteoarthritis (OA)-related alterations in the synovial fluid (SF) on friction, patient-specific SF was used as lubricant. Friction coefficients were derived from the first (μ_0_) and final 60 ​s (μ_end_) of testing.

**Results:**

Under stance phase conditions, friction was lowest at the beginning of testing (μ_0_ ​= ​0.021), but increased the most (+276 %, μ_end_ ​= ​0.079) compared to low (+47 %) and moderate loading regimes (+31 %). Under swing phase conditions low friction was maintained over time (+0 %, μ_0_ ​= ​0.041, μ_end_ ​= ​0.041).

**Conclusion:**

The friction properties of degenerated cartilage samples indicated a strong dependency on the loading regime, whereby prolonged stance phase loading led to the highest time-dependent increase in friction. Moreover, our data suggested that osteoarthritic synovial fluid was sufficient to provide low cartilage friction.

## 1 Introduction

Cartilage friction is thought to be involved in the pathogenesis and progression of osteoarthritis (OA). A scenario linking friction and OA progression was described by Lin and Klein [1]: Higher friction of otherwise healthy cartilage leads to the upregulation of catabolic enzymes. Consequently, the cartilage matrix is degraded and the surface becomes rougher. Higher friction resulting from this will in turn cause the production of more cartilage-degrading enzymes [1,2]. In this way, a friction-induced, self-enforcing vicious cycle might promote OA progression [1,3]. It is well established that cartilage friction is highly dependent on the loading conditions [[4], [5], [6]], because joint loading determines distinct lubrication mechanisms that guide the frictional properties [7]. While the interstitial fluid pressurization (IFP) lubrication relies in the biphasic nature of the cartilage, boundary lubrication involves the interaction between cartilage and synovial fluid molecules [[7], [8], [9]]. From a tribological perspective, it is therefore of interest to assess the frictional properties of osteoarthritic cartilage under various loading scenarios in order to ascertain loading regimes in a physiologic range that might elicit the highest friction. Friction coefficients of human osteoarthritic cartilage have already been evaluated under various contact stresses (0.1–0.9 ​MPa) and testing velocities (0.5–80 ​mm/s) [[10], [11], [12]]. However, existing studies differed in the lubricant used during the ex-vivo friction tests. To date, phosphate-buffered saline (PBS), healthy bovine serum or synthetic synovial fluid (sSF) [[10], [11], [12]]) were used. Because the lubricant has a significant effect on cartilage friction [6,9], deriving the load dependency of the tissue's frictional properties in OA based on literature is limited. In osteoarthritic patients, it is not only the cartilage that is affected by degradation, but also the composition of SF [9]. The concentration of its key lubricating molecules hyaluronic acid and lubricin is reduced [13,14]. The resulting compromised SF lubricity can effect cartilage friction [9] and thus, may also be involved in the hypothesized friction induced progression of OA [7]. However, we identified a lack of tribological studies on human cartilage samples that use osteoarthritic SF during friction testing. The aim of our study was to quantify the friction coefficients of degenerated human cartilage samples under four different loading regimes in a physiologically relevant range in order to identify those regimes that result in the highest friction. Thereby, we considered the effect of potential osteoarthritic changes in the SF on cartilage friction by using patient-specific synovial fluid as lubricant in our experiments.

## 2 Materials and methods

### 2.1 Sample preparation

**Fig. 1:**
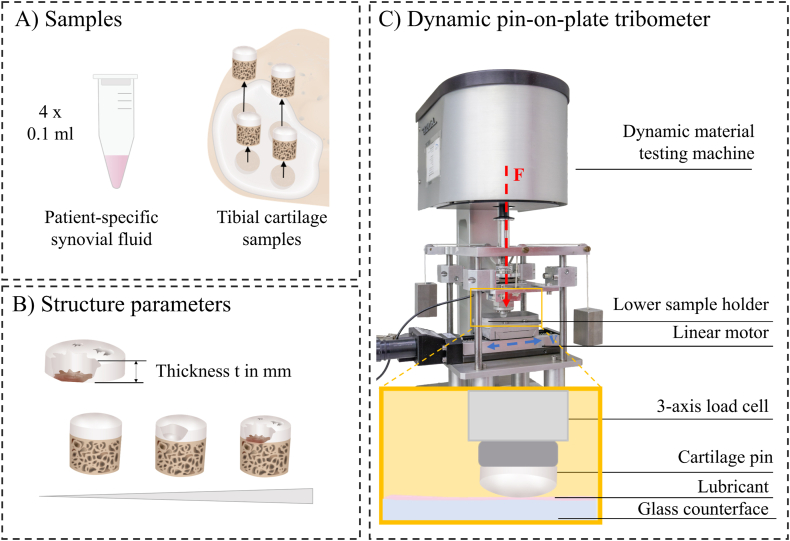
Schematic representation of the study design. A) Four cylindrical cartilage samples with a diameter of 6 ​mm were extracted from each lateral tibial plateau: three from locations under the meniscus and one at the cartilage-to-cartilage contact area. 0.1 ​ml patient specific synovial fluid was used as lubricant in each test. B) The cartilage thickness (t in mm) and the macroscopic degeneration grade according to the International Cartilage Repair Society (ICRS) classification system were determined as structural parameters prior to testing [11,15]. C) Friction experiments were performed in an established tribometer in a cartilage against glass configuration [16]. A constant axial force (F in N) was applied to the cartilage pin by the material testing maschine, while a linear motor moved the glass counterface relative to the pin at a constant velocity (v in mm). Force data were recorded by a 3-axis load cell.

According to an a-priori sample size calculation (G∗Power 3.1.9.7, effect size d ​= ​2.42, α error ​= ​0.05, power (1 ​− ​β) ​= ​0.8, [10]), six human lateral tibial plateaus and patient-specific synovial fluid (SF) were obtained from patients undergoing total knee replacement surgery due to end-stage medial gonarthrosis (72 ​± ​6 years; three male, three female; Kellgren-Lawrence: 3–4; Institutional review board approval no. 146/21 Ulm University, Germany). Immediately after surgery, the tibial plateaus were immersed in PBS and stored frozen at −20 ​°C. Each patient specific SF sample (minimum volume: 1.6; maximum volume: 2.3 ​ml) was aliquoted into 4 0.1 ​ml volumes which were stored at −80 ​°C until testing. All experiments were performed within twelve weeks after sample collection. From each plateau (N ​= ​6 biological replicates), a total of four osteochondral cores (4 technical replicates) were extracted using a trephine drill (Ø 6 ​mm), resulting in a total sample size of n ​= ​24. The sample extraction was performed at three locations under the meniscus (anterior horn, pars intermedia, posterior horn) and at the cartilage-to-cartilage contact area (Fig. 1 A). Then, the bony part or the osteochondral core was removed by cutting the cartilage along the tidemark parallel to the surface using a microtome blade. The thickness of the resulting cartilage samples (t in mm) was measured using a digital caliper (Kunzer, Forstinning, Germany, accuracy ​± ​0.05 ​mm). For fixation in the tribometer, the cut side of sample was fixed on a pin with instant adhesive. Before friction testing, the macroscopic degeneration severity of each sample was graded in accordance with the International Cartilage Repair Society (ICRS) classification system [15]. The mean value of two independent observers with minimum two years of experience in cartilage research was used.

### 2.2 Friction experiments

The frictional properties of the cartilage samples were investigated in an established dynamic pin-on-plate tribometer [16] (Fig. 1 B). The system comprises a dynamic material testing machine (ElectroForce 5500, Bose/TA Instruments, USA) for axial load application, equipped with a linear motor (VT-75, PI miCos GmbH, Germany) to enable translatory motion. The pin with the cartilage sample was screwed in a pin holder in the tribometer, whereby the in-vivo sliding direction was considered. The pin holder was attached to a 3-axis load cell used for normal and friction force acquisition (K3D40, ME Meβsysteme GmbH, Germany). All cartilage samples were tested against a glass microscope slide (VWR International LLC, USA) which was fixed in a lower sample holder (unconfined test conditions) (Fig. 1). A total of four loading profiles were applied to each cartilage sample in random order (Table 1). These loading regimes were chosen because they were defined as physiologically relevant in previous ex-vivo friction studies on degenerated human knee joint cartilage representing different types of joint loading during daily activities. Loading regime I reflected stance phase loading with a velocity (v) of 40 ​mm/s and a high contact stress (P) of 0.9 ​MPa [11,16]. Loading regime II was derived from the swing phase loading during gait, with v ​= ​80 ​mm/s and P ​= ​0.2 ​MPa [11,16]. Loading regimes III (v ​= ​0.5 ​mm/s, P ​= ​0.1 ​MPa [12]) and IV (v ​= ​1 ​mm/s, P ​= ​0.5 ​MPa [10]) consisted of low and moderate loading with low velocities, respectively. These low-intensity activities may include cycling with low resistance or passive, non-weight bearing range-of-motion exercises performed during physiotherapy [17,18]. After each test run, the cartilage sample was removed from the tribometer and immersed in PBS for 10 ​min to allow for tissue relaxation under free swelling conditions. A marking of the pin's position in the tribometer allowed for consistent orientation of the sample in all test runs. In each test, the target axial force was applied by the material testing machine within 5 ​s and maintained constant for a test duration of 10 ​min. This duration was selected because previous research indicated that nearly half of people with knee OA did not engage in physical activity more than 10 ​min [19]. Once the target force was reached, the motor moved the glass surface relative to the pin with the target velocity and force data acquisition was started (100 ​Hz). Each test was lubricated with 0.1 ​ml of patient-specific SF matching the respective cartilage sample (open bath with minimal fluid, no multiple use of SF samples). This volume was sufficient to keep the cartilage surface constantly covered throughout the 10-min testing time.

**Table 1:** Test parameters (contact stress P in MPa, velocity v in mm/s) of the four investigated loading regimes (I–IV). Glass was used as a friction partner in all experiments. Each sample was lubricated with 0.1 ​ml patient-specific synovial fluid.

Loading regime	I	II	III	IV
P in MPa	0.9	0.2	0.1	0.5
v in mm/s	40	80	0.5	1
Friction partner	Glass			
Lubricant	0.1 ​ml patient-specific synovial fluid			

The stroke length of one cycle was 60 ​mm, which was previously defined as the distance the cartilage surfaces in the knee move relative to each other during gait [16]. Friction was quantified according to Coulomb's law (μ ​= ​friction force in N/normal force in N) at the beginning of the test by averaging the first 60 ​s of testing (μ_0_: mean value of 6000 data points) and the last 60 ​s of testing (μ_end_: mean value of 6000 data points) (see highlighted areas in Fig. 4) using a custom MATLAB script (MATLAB R2024a, The MathWorks Inc., Natick, United States). Data at the reversal points of the cyclic motions, where the motor stops and makes a change of direction, were not included in the calculation of μ.

### 2.3 Statistics

Statistical analyses were performed using Graph Pad Prism 10 (GraphPad, San Diego, CA).

Kruskal-Wallis testing was applied to identify possible differences in friction between samples with ICRS 1, ICRS 2 and ICRS 3. Because no significant differences were found, we pooled the results of all tested samples, resulting in a total sample size of n ​= ​24 for statistical analyses.

Shapiro-Wilk testing indicated non-normally distributed data. Wilcoxon testing was applied to compare μ_0_ and μ_end_ under each loading regime. Differences in friction between the different loading profiles were analyzed using Friedman test with Dunn's post-hoc testing, while p ​≤ ​0.05 was considered to be statistically significant.

## 3 Results

**Fig. 2:**
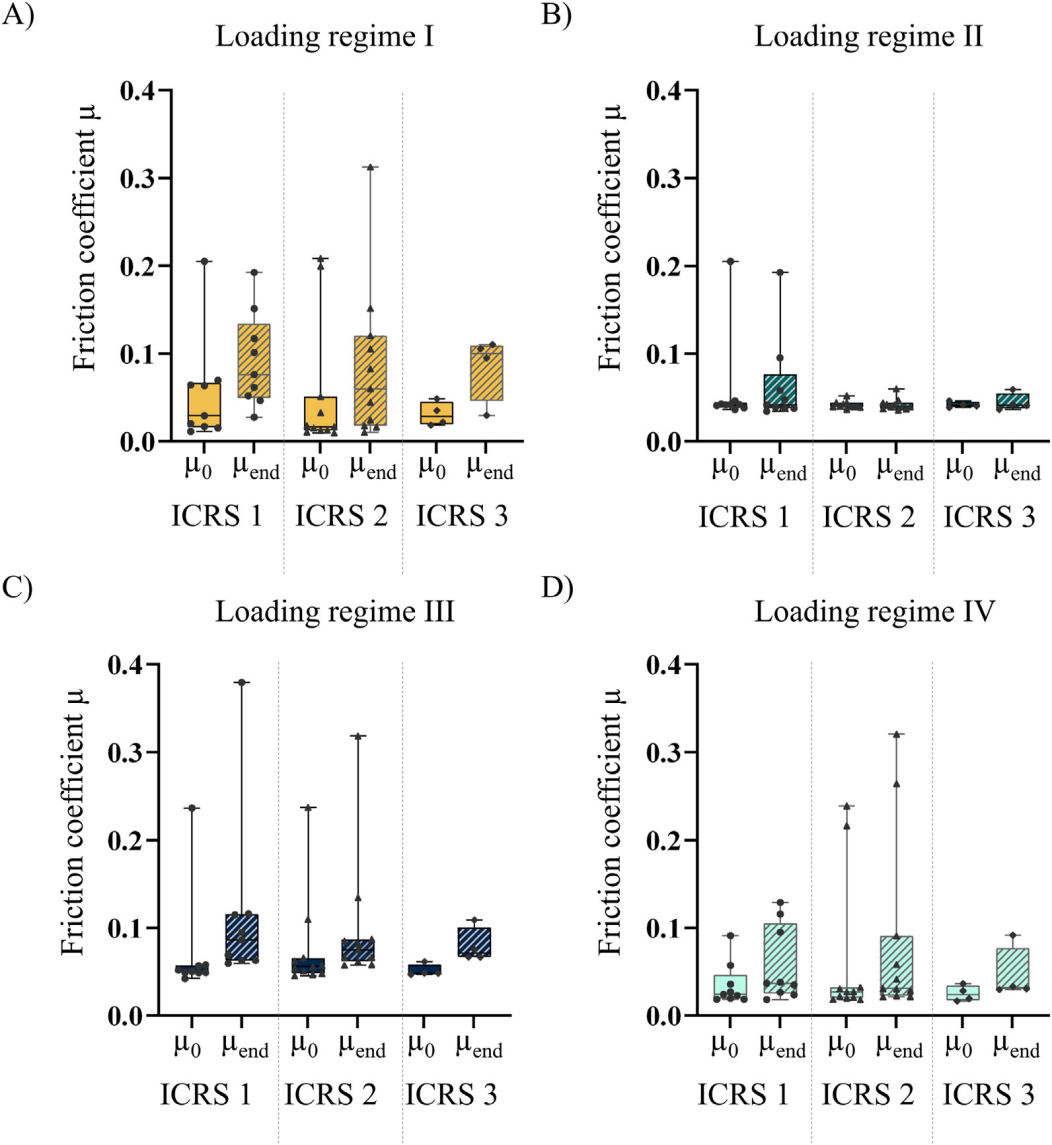
Results of the friction experiments by degeneration severity shown as box plot of μ_0_ and μ_end_ under A) loading regime I, B) loading regime II, C) loading regime III and D) loading regime IV. Samples per degeneration grade: ICRS 1: n ​= ​9, ICRS 2: n ​= ​11, ICRS 3: n ​= ​4. p ​> ​0.05.

The cartilage samples indicated a median thickness of 2.25 ​mm (95 % CI: [1.86, 2.672]). The ICRS scores ranged between 1 and 3, whereby 9/24 (37 %) samples indicated ICRS 1, 11/24 ICRS 2 (46 %) and 4/24 ICRS 3 (17 %). The comparison of friction coefficients of samples with ICRS 1, ICRS 2 and ICRS 3 revealed no significant differences neither for μ_0_ nor for μ_end_ in any of the applied loading regimes (Fig. 2).

**Fig. 3:**
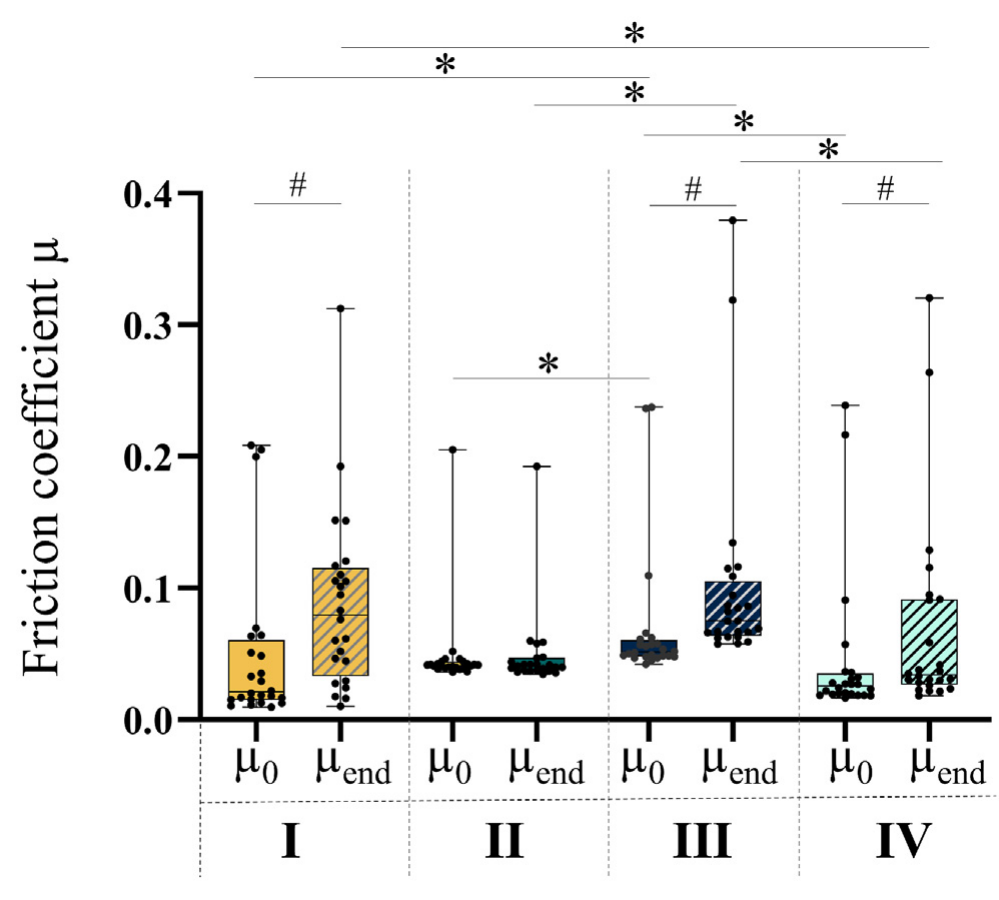
Results of the friction coefficients μ_0_ and μ_end_ shown as box plot of n ​= ​24 tested samples (N ​= ​6 biological replicates x 4 technical replicates) under loading regime I [16] (velocity (v) ​= ​40 ​mm/s, contact stress (P) ​= ​0.9 ​MPa), II [16] (v ​= ​80 ​mm/s, P ​= ​0.2 ​MPa), III [12] (v ​= ​0.5 ​mm/s, P ​= ​0.1 ​MPa) and IV [10] (v ​= ​1 ​mm/s, P ​= ​0.5 ​MPa. n ​= ​24, # ​= ​p ​< ​0.05: Wilcoxon non-parametric test, ∗ ​= ​p ​< ​0.05 Friedman non-parametric test with multiple comparison.

**Fig. 4:**
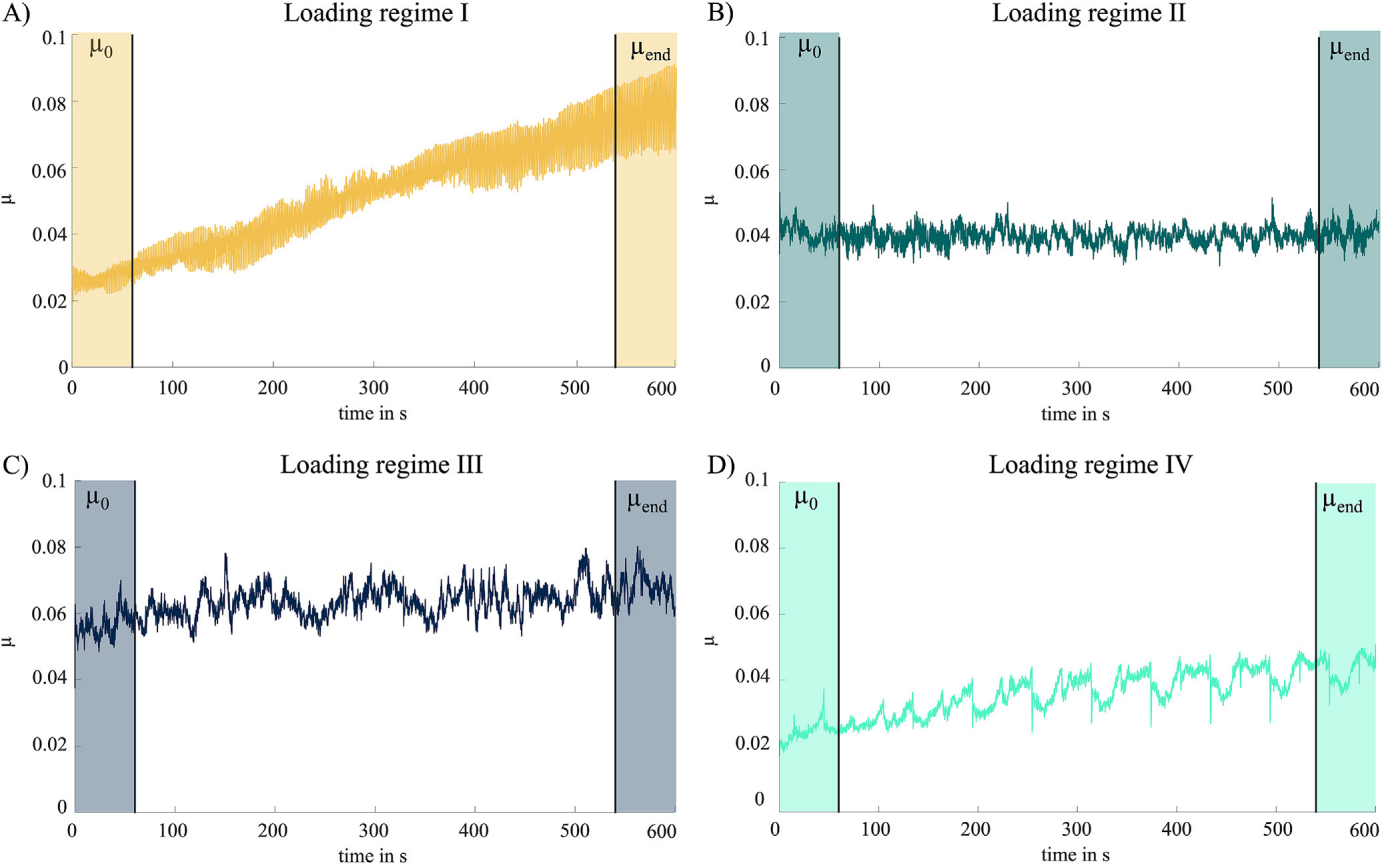
Time-dependent evolution of the friction coefficient. The data are shown as the median of all specimens calculated at each recorded time point under A) loading regime I, B) loading regime II, C) loading regime III, D) loading regime IV). In each graph, the data of the first and last 60 ​s are highlighted to visualize which data were included in the calculation of μ_0_ and μ_end_ (averaging the first 6000 and last 6000 data points, performed for individual time-series data of each sample).

Wilcoxon testing revealed that μ_end_ was significantly higher compared to μ_0_ under all tested loading regimes, except under loading regime II (Fig. 3). The highest increase in friction over time was observed under loading regime I (+276 %) (Fig. 4). A time-dependent increase in the friction coefficient of approximately 47 % and 31 % were found under loading regime III and IV, respectively. The comparison between the different loading regimes revealed that μ_0_ under loading regime III was significantly higher compared to μ_0_ under loading regimes I, II and IV, respectively. The comparison of μ_end_ between the different loading regimes revealed significant differences between loading regimes I and IV, between II and III and between III and IV.

## 4 Discussion

The results of our study revealed a significant impact of the applied loading regime on the ex-vivo friction coefficients of degenerated human cartilage samples. Key factors in ex-vivo tribological tests are the applied testing velocity, the contact pressure and the lubricant as they significantly regulate different lubrication regimes and thus, friction [1,3,9,20]. Interestingly, μ_0_ under stance phase loading (regime I with the highest contact stress of 0.9 ​MPa) was lowest compared to the loading regimes with low or moderate contact stress. This might be explained by the interstitial fluid pressurization (IFP) lubrication: Higher loading of the tissue causes higher pressure of the matrix's interstitial fluid, which allows the fluid phase to bear a larger portion of the applied contact stress, resulting in lower friction [21]. Our findings indicated that, in the initial stance phase (heel-on), the osteoarthritic cartilage is able to promote sufficient IFP lubrication necessary to minimize friction under stance phase loading. However, friction under stance phase loading in profile I displayed the greatest increase in friction over the testing time of 10 ​min. On the basis of previous observations, we expected an increase in friction over time, when testing cartilage against glass, because constant loading leads to exudation of the interstitial fluid [22]. As a result, the IFP lubrication gets compromised while the dominant lubrication mechanism becomes boundary lubrication, which is generally characterized by higher friction [21]. In this regime, friction is regulated by boundary lubrication molecules like hyaluronic acid and lubricin, both present in the SF and at the cartilage surface. These molecules are reduced in osteoarthritic patients [13], which might contributed to even higher friction. Together, our data let suggest that particularly prolonged stance phase loading could not promote sufficient lubrication conditions over time, as a friction increase of 276 ​% was observed under loading regime I. Short joint loading periods in combination with weight loss to reduce joint contact stress during the stance phase of gait would probably be beneficial to reduce cartilage friction in OA [23]. However, it can be inferred from the results obtained under loading regime III that the contact stress may not be the only friction-regulating factor. Although the applied contact stress was lowest under regime III (0.1 ​MPa), μ_0_ was highest within the first 60 ​s of testing. Furthermore, μ_end_ reached a similar μ_end_ value as under demanding stance phase loading under regime I. It is reasonable to assume that the combination of low contact stress and low velocity could not promote efficient lubrication conditions. Possible reasons include that the contact stress was too low for the interstitial fluid to initially generate an adequate pressure for sufficient IFP lubrication, thus explaining the high μ_0_ values under loading regime III. In addition, the velocity could have been too low to provide sufficient IFP over time, which is by theory maintained as long as the migration speed is much greater than the time needed for the fluid to flow out of the cartilage matrix [10]. Furthermore, the non-Newtonian SF is most effective as a lubricant at moderate to high sliding velocities, typically between 1 ​mm/s to several mm/s (up to 80 ​mm/s) [1,9]. Indeed, the other investigated loading regimes consisted of velocities of 1 ​mm/s or faster and displayed lower friction coefficients within the first 60 ​s of testing compared to those of loading regime III. Kupratis et al. hypothesized that for compromised tissues like degenerated cartilage, high movement velocities are beneficial to promote sufficient lubrication conditions and accordingly low friction [23]. The reason is that osteoarthritic cartilage is characterized by increased permeability, which in turn causes the interstitial fluid to subside more rapidly than in healthy cartilage [10,23]. Consequently, a higher migration speed is needed to maintain a friction-minimizing IFP [23]. Our results under loading regime II confirmed that increased articulation speeds contribute to a low friction coefficient in osteoarthritic cartilage. Interestingly, loading profile II, with the highest testing velocity of 80 ​mm/s, resulted in initially low friction, which moreover, did not increase over time. It is well documented that cartilage friction decreases as sliding velocity increases [4,24]. Moore and Burris introduced the concept of tribological rehydration [25], which describes a sliding-induced recovery of the IFP under stationary contact conditions, particularly effective under high sliding speeds (>30 mm-1) [5,26]. It might be that the high velocity of 80 ​mm/s in loading regime II provided effective, sliding-induced lubrication conditions which prevented an increase in friction over time. In addition, the high sliding velocity may have increased fluid entrainment at the leading edge of the cylindrical sample [27,28]. Compared to the other loading regimes with lower velocity, this might have generated higher hydrodynamic pressures, which allowed to maintain friction over time. Together, these hydrodynamic phenomena [5] might explain why friction did not increase over time under swing phase loading.

When comparing the present friction coefficients with those reported in previous studies conducted under comparable loading regimes but with different lubricants, we found that the friction coefficients using patient-specific SF were lower than those reported for PBS or synthetic SF, but higher than those reported for healthy bovine SF (Table 2). Although the investigated loading regimes were derived from existing studies on human OA cartilage samples, our study design differed in several aspects. These include the applied OA severity scoring system, the determination timepoint of μ and the amount of lubricant used [[10], [11], [12]]. All of these factors are likely to influence ex-vivo cartilage friction, thus limiting a detailed comparison with literature values. Such methodological differences make it difficult to derive the load dependency of the tissue's frictional properties in OA based on literature. Thus, one advantage of our study design was that it allowed a direct comparison of the OA cartilage's frictional properties under different loading regimes. Moreover, by using patient-specific SF we simulated conditions which represent the osteoarthritic knee environment, which we believe is a decisive factor for the transferability and interpretability of in-vitro findings to the clinical situation. However, this study is not without limitations. First, the frictional properties were quantified in a cartilage against glass configuration. It has been shown that the tissue's frictional properties in a cartilage against cartilage configuration differ from those obtained when testing cartilage against glass [11]. However, the tribological system cartilage against glass was chosen because it provides a standardized friction partner, which allowed for the elimination of one influencing factor while investigating the complex tribological properties of cartilage ex vivo. To further advance the understanding of friction in OA, future studies should also consider the entire (patho-) physiologic knee environment. This would entail testing cartilage against cartilage lubricated with patient-specific SF. Second, the study does not allow for a more comprehensive analysis of different lubrication mechanisms, as cartilage thickness was only measured prior to friction testing. Data of the cartilage thickness during testing or immediately afterwards would have offered valuable insights into fluid-related mechanism such as IFP lubrication or sliding-induced tribological rehydration [5,8,28,29]. Furthermore, the composition of the synovial fluid, specifically the presence of lubricating molecules such as hyaluronic acid and lubricin, could not be analyzed due to limited SF volume. This would have offered more insights into boundary lubrication effects. Third, we did not investigate healthy human cartilage samples because it was not possible to obtain synovial fluid samples from young body donors stored at −80 ​°C, a temperature that preserves the SF's biochemical and biophysical properties [30]. Therefore, we could not evaluate whether the load dependency of cartilage friction is different in OA compared to healthy conditions. The scope of the presented study was to evaluate the tribological functionality of degenerated cartilage under different loading regimes by determining the friction coefficients. In order to build on the findings presented here, future research should also include additional important tribological aspects like cartilage wear characterization and synovial fluid properties.

**Table 2:** Previously published friction coefficients of degenerated human cartilage. μ values and applied lubricants reported in previous studies from which the applied loading profiles were derived are summarized.

	I	II	III	IV	
Literature values of μ	0.03–0.17^a^ [11]	0.05–0.27^a^ [11]	0.11–0.26^a^ [12]	0.02–0.13 [10]	0.01–0.04 [10]
Used lubricant	sSF	sSF	PBS	PBS	Bovine SF
Loading profile	Gait cycle with stance and swing phase		0.1 ​MPa 0.5 ​mm/s	0.5 ​MPa 1 ​mm/s	

a Values are extracted from graphs using WebPlotDigitizer; SF ​= ​synovial fluid, sSF ​= ​synthetic synovial fluid, PBS ​= ​phosphate buffered saline.

Whether or not cartilage friction is altered in OA remains inconclusive. In the present study, cartilage friction with patient-specific SF did not show a dependency on macroscopic OA severity. While our findings here are in agreement with those of Caligaris et al. [10], both, de Roy et al. [11] and Neu et al. [12] did report that cartilage friction increased with progressing degeneration. Different OA severity assessment methods might be one reason for the contradictory outcomes of these studies. A consensus on OA scoring methods applied in ex-vivo tribological studies might be one option to improve the comparability between studies.

In conclusion, the present study provides novel insights into the frictional properties of degenerated cartilage across various loading regimes under patient-specific lubrication conditions. Identifying loading regimes in a physiologic range that cause the highest friction is of interest in the context of the postulated association between friction and OA, in which it is assumed that cartilage degradation might be initiated and progressed by a friction-induced upregulation of catabolic enzymes [1].

## Author contribution statement

LdR: Conceptualization, Project Administration, Formal Analysis, Visualization, Writing- Original Draft Preparation.

JWM: Investigation, Writing – Review and Editing.

MF: Resources, Writing – Review and Editing.

AI: Supervision, Funding Acquisition, Writing – Review and Editing.

AMS: Project Administration, Supervision, Funding Acquisition, Writing – Review and Editing.

## Conflict of interest

The authors have no conflict of interest.

## References

[1] Lin W., Klein J. (2021). Recent progress in cartilage lubrication. Adv. Mater..

[2] Desrochers J., Amrein M.W., Matyas J.R. (2013). Microscale surface friction of articular cartilage in early osteoarthritis. J. Mech. Behav. Biomed. Mater..

[3] Link J.M., Salinas E.Y., Hu J.C., Athanasiou K.A. (2020). The tribology of cartilage: mechanisms, experimental techniques, and relevance to translational tissue engineering. Clin. Biomech..

[4] Gleghorn J.P., Bonassar L.J. (2008). Lubrication mode analysis of articular cartilage using Stribeck surfaces. J. Biomech..

[5] Farnham M.S., Ortved K.F., Burris D.L., Price C. (2021). Articular cartilage friction, strain, and viability under physiological to pathological benchtop sliding conditions. Cell. Mol. Bioeng..

[6] Furmann D., Nečas D., Rebenda D., Čípek P., Vrbka M., Křupka I. (2020). The effect of synovial fluid composition, speed and load on frictional behaviour of articular cartilage. Materials.

[7] Rajankunte Mahadeshwara M., Al-Jawad M., Hall R.M., Pandit H., El-Gendy R., Bryant M. (2024). How do cartilage lubrication mechanisms fail in osteoarthritis? A comprehensive review. Bioengineering.

[8] Farnham M., Ortved K., Horner J., Wagner N., Burris D.L., Price C. (2021). Lubricant effects on articular cartilage sliding biomechanics under physiological fluid load support. Tribol. Lett..

[9] Marian M., Shah R., Gashi B., Zhang S., Bhavnani K., Wartzack S. (2021). Exploring the lubrication mechanisms of synovial fluids for joint longevity - a perspective. Coll. Surf. B Biointer..

[10] Caligaris M., Canal C.E., Ahmad C.S., Gardner T.R., Ateshian G.A. (2009). Investigation of the frictional response of osteoarthritic human tibiofemoral joints and the potential beneficial tribological effect of healthy synovial fluid. Osteoarthr. Cartil..

[11] de Roy L., Schlickenrieder K., Rüger M., Faschingbauer M., Ignatius A., Seitz A.M. (2023). Impact of degeneration and material pairings on cartilage friction: cartilage versus glass. J. Orthop. Res..

[12] Neu C.P., Reddi A.H., Komvopoulos K., Schmid T.M., Di Cesare P.E. (2010). Increased friction coefficient and superficial zone protein expression in patients with advanced osteoarthritis. Arthritis Rheum..

[13] Guenther L.E., Pyle B.W., Turgeon T.R., Bohm E.R., Wyss U.P., Schmidt T.A. (2014). Biochemical analyses of human osteoarthritic and periprosthetic synovial fluid. Proc. Inst. Mech. Eng. H.

[14] Herbster M., Nizinkovskyi R., Bollmann M., Bartel D., Lohmann C., Krüger M. (2021). Synthesis of a lubricant to mimic the biorheological behavior of osteoarthritic and revision synovial fluid. Lubricants.

[15] Dwyer T., Martin C.R., Kendra R., Sermer C., Chahal J., Ogilvie-Harris D. (2017). Reliability and validity of the arthroscopic international cartilage Repair society classification system: correlation with histological assessment of depth. Arthroscopy.

[16] Warnecke D., Meßemer M., Roy L., Stein S., Gentilini C., Walker R. (2019). Articular cartilage and meniscus reveal higher friction in swing phase than in stance phase under dynamic gait conditions. Sci. Rep..

[17] Sundar Doss S., Rekha K., Suganthirababu P. (2014). Effects of non weight bearing strength training for knee osteoarthritis. Int. J. Res. Pharm. Sci..

[18] Kutzner I., Heinlein B., Graichen F., Rohlmann A., Halder A.M., Beier A. (2012). Loading of the knee joint during ergometer cycling: telemetric in vivo data. J. Orthop. Sports Phys. Ther..

[19] Dunlop D.D., Song J., Semanik P.A., Chang R.W., Sharma L., Bathon J.M. (2011). Objective physical activity measurement in the osteoarthritis initiative: are guidelines being met?. Arthr. Rheum..

[20] Li Y., Yuan Z., Yang H., Zhong H., Peng W., Xie R. (2021). Recent advances in understanding the role of cartilage lubrication in osteoarthritis. Molecules.

[21] Ateshian G.A. (2009). The role of interstitial fluid pressurization in articular cartilage lubrication. J. Biomech..

[22] Caligaris M., Ateshian G.A. (2008). Effects of sustained interstitial fluid pressurization under migrating contact area, and boundary lubrication by synovial fluid, on cartilage friction. Osteoarthr. Cartil..

[23] Kupratis M.E., Gure A.E., Benson J.M., Ortved K.F., Burris D.L., Price C. (2022). Comparative tribology II-Measurable biphasic tissue properties have predictable impacts on cartilage rehydration and lubricity. Acta Biomater..

[24] Gleghorn J.P., Doty S.B., Warren R.F., Wright T.M., Maher S.A., Bonassar L.J. (2010). Analysis of frictional behavior and changes in morphology resulting from cartilage articulation with porous polyurethane foams. J. Orthop. Res..

[25] Moore A.C., Burris D.L. (2017). Tribological rehydration of cartilage and its potential role in preserving joint health. Osteoarthr. Cartil..

[26] Elkington R.J., Hall R.M., Beadling A.R., Pandit H., Bryant M.G. (2024). Engineering tribological rehydration of cartilage interfaces: assessment of potential polyelectrolyte mechanisms. Tribol. Int..

[27] Burris D.L., Moore A.C. (2017). Cartilage and joint lubrication: new insights into the role of hydrodynamics. Biotribology.

[28] Burris D.L., Ramsey L., Graham B., Price C., Moore A. (2019). How sliding and hydrodynamics contribute to articular cartilage fluid and lubrication recovery. Tribol. Lett..

[29] Schwarz M.L., Reisig G., Schneider-Wald B., Weiss C., Hauk L., Schütte A. (2022). Coefficient of friction and height loss: two criteria used to determine the mechanical property and stability of regenerated versus natural articular cartilage. Biomedicines.

[30] Jaggard M.K.J., Boulangé C.L., Graça G., Akhbari P., Vaghela U., Bhattacharya R. (2021). The influence of sample collection, handling and low temperature storage upon NMR metabolic profiling analysis in human synovial fluid. J. Pharmaceut. Biomed. Anal..

